# Dynamic Locomotor Capabilities Revealed by Early Dinosaur Trackmakers from Southern Africa

**DOI:** 10.1371/journal.pone.0007331

**Published:** 2009-10-06

**Authors:** Jeffrey A. Wilson, Claudia A. Marsicano, Roger M. H. Smith

**Affiliations:** 1 Museum of Paleontology & Department of Geological Sciences, University of Michigan, Ann Arbor, Michigan, United States of America; 2 Departamento de Ciencias Geológicas, Universidad de Buenos Aires, Buenos Aires, Argentina; 3 Department of Karoo Paleontology, Iziko South African Museum, Cape Town, South Africa; Raymond M. Alf Museum of Paleontology, United States of America

## Abstract

**Background:**

A new investigation of the sedimentology and ichnology of the Early Jurassic Moyeni tracksite in Lesotho, southern Africa has yielded new insights into the behavior and locomotor dynamics of early dinosaurs.

**Methodology/Principal Findings:**

The tracksite is an ancient point bar preserving a heterogeneous substrate of varied consistency and inclination that includes a ripple-marked riverbed, a bar slope, and a stable algal-matted bar top surface. Several basal ornithischian dinosaurs and a single theropod dinosaur crossed its surface within days or perhaps weeks of one another, but responded to substrate heterogeneity differently. Whereas the theropod trackmaker accommodated sloping and slippery surfaces by gripping the substrate with its pedal claws, the basal ornithischian trackmakers adjusted to the terrain by changing between quadrupedal and bipedal stance, wide and narrow gauge limb support (abduction range = 31°), and plantigrade and digitigrade foot posture.

**Conclusions/Significance:**

The locomotor adjustments coincide with changes in substrate consistency along the trackway and appear to reflect ‘real time’ responses to a complex terrain. It is proposed that these responses foreshadow important locomotor transformations characterizing the later evolution of the two main dinosaur lineages. Ornithischians, which shifted from bipedal to quadrupedal posture at least three times in their evolutionary history, are shown to have been capable of adopting both postures early in their evolutionary history. The substrate-gripping behavior demonstrated by the early theropod, in turn, is consistent with the hypothesized function of pedal claws in bird ancestors.

## Introduction

**Figure 1:**
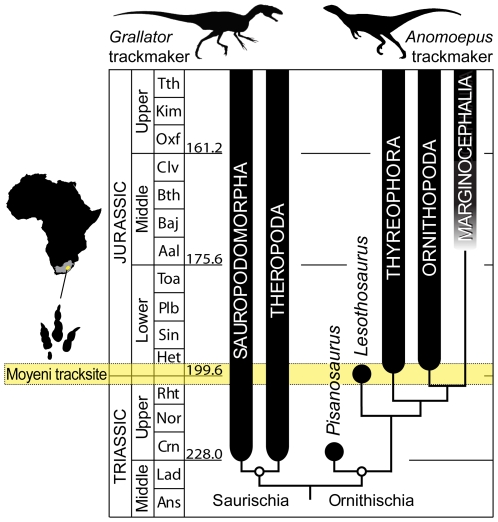
The Moyeni trackway locality and its stratigraphic and phylogenetic context. The thumbnail map of Africa shows areal extent of main Karoo Basin (grey) and the country of Lesotho (yellow). The phylogeny depicts the basic interrelationships of major dinosaur clades [41] on a timescale. Subsequent analyses have resolved *Lesothosaurus* at the base of Thyreophora [45], but most agree that it is positioned near the base of clade uniting Thyreophora, Ornithopoda, and Marginocephalia. The Moyeni tracksite (marked by yellow band) preserves tracks made by early dinosaur trackmakers, well after the initial divergence of saurischians and ornithischians (early Late Triassic) but well before the origin of flight (Upper Jurassic) and prior to three independent acquisitions of quadrupedal posture (Early–Middle Jurassic) [41], [42]. The graded stratigraphic range for Marginocephalia reflects uncertainty in its first appearance date; icons atop diagram are representative theropod and ornithischian dinosaurs [41].

The earliest dinosaurs were small, bipedal forms that are first recorded ca. 228 million years ago in the Late Triassic, when the Earth's continental landmasses were interlocked as Pangea [1]. Already present in these earliest dinosaur-bearing sediments are two basal lineages, Ornithischia and Theropoda, both of whose locomotor evolution would diverge dramatically from the ancestral dinosaurian condition during the Jurassic [2]. The ornithischian line gave rise to three different lineages, each capable of quadrupedal locomotion, during the Early and Middle Jurassic (ca. 200–161 Ma), and the theropod line gave rise to birds capable of powered flight by the close the Late Jurassic (145 Ma). We report new evidence from an Early Jurassic tracksite in southern Africa [3] that provides a rare glimpse at dynamic locomotor capabilities of these two dinosaur lineages at an early phase in their evolution (Fig. 1). These dynamic capabilities, which are not directly apparent from the body fossil record, imply a greater locomotor plasticity in early ornithischians than in early theropods and bring to light a previously unknown functionality in theropods.

### Moyeni Tracksite, Lesotho

Extensive, well-preserved fossil tetrapod trackways of the Stormberg Group in the Karoo Basin of southern Africa provide significant information about faunal diversity and turnover during the early Mesozoic in southern Pangea [4]–[6]. Many of these trackways occur in erosion-resistant sandstones of the Elliot Formation that are positioned near the Triassic-Jurassic boundary (199.6 Ma; [7]). Perhaps the most spectacular is the Moyeni tracksite in southern Lesotho, which was discovered and first described in detail by Paul Ellenberger [8]–[10]. The Moyeni tracksite is generally considered to be Early Jurassic in age [6], but uncertainty about the exact position of the Triassic–Jurassic boundary within the Elliot Formation precludes more specific temporal resolution.

Originally, Ellenberger [3] described 13 different track-types at Moyeni, all of which he considered endemic to the Karoo Basin, and provided detailed reconstructions of the behavior implied by many of the longer trackways. A critical influence on Ellenberger's reconstructions of trackmaker behavior was his interpretation of the depositional environment as an emergent, elongated sandbank formed at the mouth of a river flowing into a large lake. Consequently, he interpreted many of the tracks we discuss herein as having been made by swimming dinosaurs that had webbed hind feet (see Text S1).

**Figure 2:**
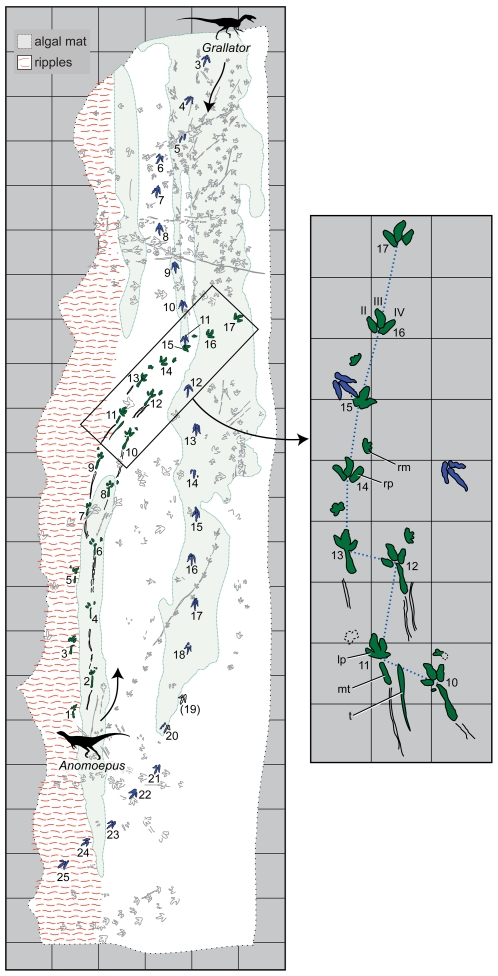
Map of major geological features and tracks at the Moyeni tracksite. Trackways of *Anomoepus* and *Grallator* are in solid green and blue, respectively; all other trackways are in 50% grey. Trackway numbering (Arabic numerals) begins with the first recognized step of each trackmaker. *Grallator* tracks 1 and 2 are not shown because they are now underneath a retaining wall [3]. Red wavy lines indicate ripple marks; solid light grey fields indicate algal-matted surface. The inset highlights postural and gait changes in the *Anomoepus* trackway, which was made by a basal ornithischian. A shift from wide-gauge to narrow-gauge posture (between tracks 13 and 14) is marked by shift in pace angulation (blue dotted line) and was accompanied by a brief pause, during which the tail registered on the substrate when tracks 12 and 13 were impressed. The shift from a quadrupedal to a bipedal gait (between tracks 15 and 16) occurs atop the algal matted surface. Note that *Grallator* track 11 (blue) overprints *Anomoepus* track 15, indicating it was made later. Grid pattern forms 1 meter squares for both maps. Abbreviations: lm, left manus; lp, left pes; mt, metatarsus; rm, right manus; rp, right pes; t, tail; Roman numerals indicate pedal digits.

The Moyeni tracksite records more than 250 tetrapod footprints and associated invertebrate traces on a 100 m^2^ sandstone surface (Fig. 2). The remarkable richness and level of detail preserved at the Moyeni tracksite offer a unique opportunity to investigate the locomotor habits of early dinosaurs.

### Paleoenvironmental Interpretation

We interpreted the sedimentary sequence below and above the track-bearing surface as having accumulated on the inside bank of a meander loop rather than in a lacustrine setting, with the main trackway surface preserved on one of several scroll bars making up a low-angle point bar [11]. There, fossil trackways probably were imprinted over the course of days or weeks on the sandy inner bank of a channel meander, recording a brief moment in a southern African terrestrial ecosystem. The trackway surface has three distinctive sections that differ in their slope and surface texture: a lower, ripple-marked portion; a smooth bar slope inclined at approximately 20°; and a relatively flat bar top with a distinctive algal-matted texture (Fig. 2). Tracks cover nearly the entire area, but they are most abundant and best preserved on the algal-matted top surface. Here we focus on the two longest trackways, which cross all three surfaces, and the trackmakers' dynamic responses to differences in slope and slipperiness.

### Ichnotaxonomy and Trackmaker Identification

Although Ellenberger [3] recognized similarities with tracks described from the Newark Supergroup [12], [13], he created 7 new ichnogenera and 13 new ichnospecies to encompass the track diversity at Moyeni. The two dinosaur track-types that we will focus on in this contribution were named *Neotrisauropus deambulator* and *Moyenisauropus natator* [3](Text S1). Five other ichnospecies were named for the latter ichnogenus and distinguished by somewhat subtle features—many of which may reflect behavioral and/or preservational, rather than anatomical, variation. Revisions of Ellenberger's ichnotaxonomy synonymized all but one of the Moyeni ichnotaxa, subsuming *Neotrisauropus* and *Moyenisauropus* within *Grallator* and *Anomoepus*, respectively [4], [14]–[16]. Based on our first-hand observations of the Moyeni footprints, we concur that there is little morphological distinction between the Moyeni and Newark ichnogenera or between ichnospecies of *Moyenisauropus* and apply the revised ichnogenus names here. Future taxonomic work must determine whether the various ichnospecies named by Ellenberger must also be synonymized.

The fossilized tracks and trackways at Moyeni provide direct evidence of how early dinosaurs behaved in life. However, because trackways record the interaction of soft tissues with the substrate—typically only the undersides of the manus and pes—trackmaker identifications are imprecise and rely on a combination of stratigraphic, geographic, and morphological coincidence with body fossils [17]. Despite this constraint, fossil tracks can provide powerful insight into trackmaker paleobiology. In this contribution, we use soft tissue features preserved in the tracks to identify skeletal synapomorphies diagnosing particular trackmaker subgroups [18]. This method results in coarse yet falsifiable identifications that can be used to interpret trackmaker paleobiology. Tetrapod footprints at Moyeni include tracks produced ornithischian and theropod dinosaurs (ichnogenera *Anomoepus*, *Grallator*), basal tetrapods (ichnogenus *Episcopopus*), and crurotarsal archosaurs (“chirotheroid”-type ichnotaxon) [11].

## Methods

The map in Figure 2 was made by gridding the entire trackway surface, photographing each 1 m square, and drawing all footprints at 1∶10 scale. Trackway measurements reported in Text S2 follow standard protocol, summarized in [19]. Stride length is measured as the straight-line distance between homologous points on successive footfalls of the same foot. Pace length is measured as the straight line distance between homologous points on left and right manus or pes prints; successive pace lengths (i.e., R–L–R or L–R–L) form an angle that is measured as pace angulation. Linear measurements were made to the nearest 0.5 centimeter and were measured *in situ* unless noted; angular measurements were made to the nearest 0.5 degree using a high-resolution map of the trackway surface. Some tracks are no longer accessible because they are no longer visible (e.g., beneath retaining wall) or no longer preserved. These track measurements were estimated to the nearest 1.0 cm or degree based on maps in [3]. Gauge width was measured as the straight-line distance between a foot and the midpoint between two footfalls of its opposite (i.e., the midpoint of the stride). Three-dimensional data were collected using a HandyScan HZ at 0.63–0.88 mm resolution.

## Results

**Figure 3:**
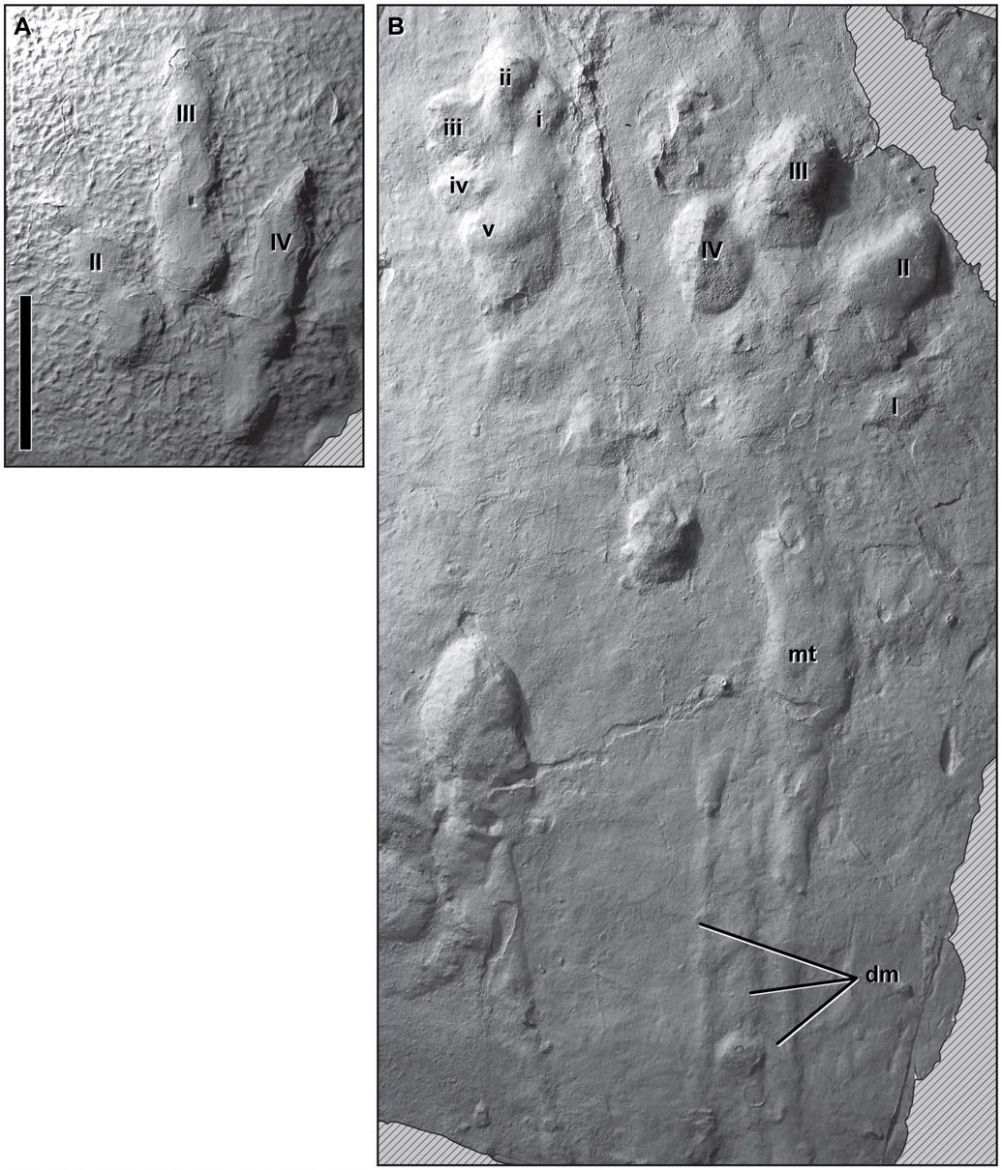
Moyeni dinosaur tracks. Photographs of plaster casts (positives) of *Grallator* track 6 (A) and *Anomoepus* track 8 (B) made at the Moyeni tracksite by the authors. Tracks are shown at the same scale (10 cm), and hatching pattern indicates broken surfaces. The *Grallator* hind foot print was made by pedal digits II–IV; the trackmaker's phalangeal formula was 3–4–5. Digits I and V did not contact the substrate. The rugose texture surrounding the print is the algal mat. The *Anomoepus* manus–pes couple registers all five manual digits (i–v), four pedal digits (I–IV), the metatarsus (mt), and toe drag marks (dm). Additional structures to the left of the pes are incidental marks made by a different trackmaker.

The two dinosaur trackmakers recorded numerous tracks and trackways at Moyeni. We focus on two lengthy trackways of *Anomoepus* and *Grallator* [3], [4] that traversed the heterogeneous surface of the point bar in opposite directions in close temporal succession, as indicated by a *Grallator* footprint that overprinted a fresh *Anomoepus* footprint as it crossed its path (Figs. 2, 3). We identify these trackmakers as dinosaurs because both trackways indicate animals capable of bipedal posture walking with a parasagittal gait on functionally tridactyl (three-toed) pes. *Grallator* can be identified more specifically as a theropod dinosaur because its tracks lack metatarsal traces, and the functionally tridactyl foot bears an elongate digit III and clearly marked, sharply pointed pedal ungual traces. *Anomoepus*, in turn, can be identified as a basal ornithischian by its symmetrical, functionally tridactyl foot bearing blunt unguals and a pentadactyl manus with subequal digits with gently rounded ends [16], [19]. The lack of sharp ungual traces in *Anomoepus* excludes basal sauropodomorphs, theropods, and heterodontosaurids as potential trackmakers, because all can be expected to create manus prints with one or more sharply defined ungual traces.

### Theropod Trackmaker (*Grallator*)

**Figure 4:**
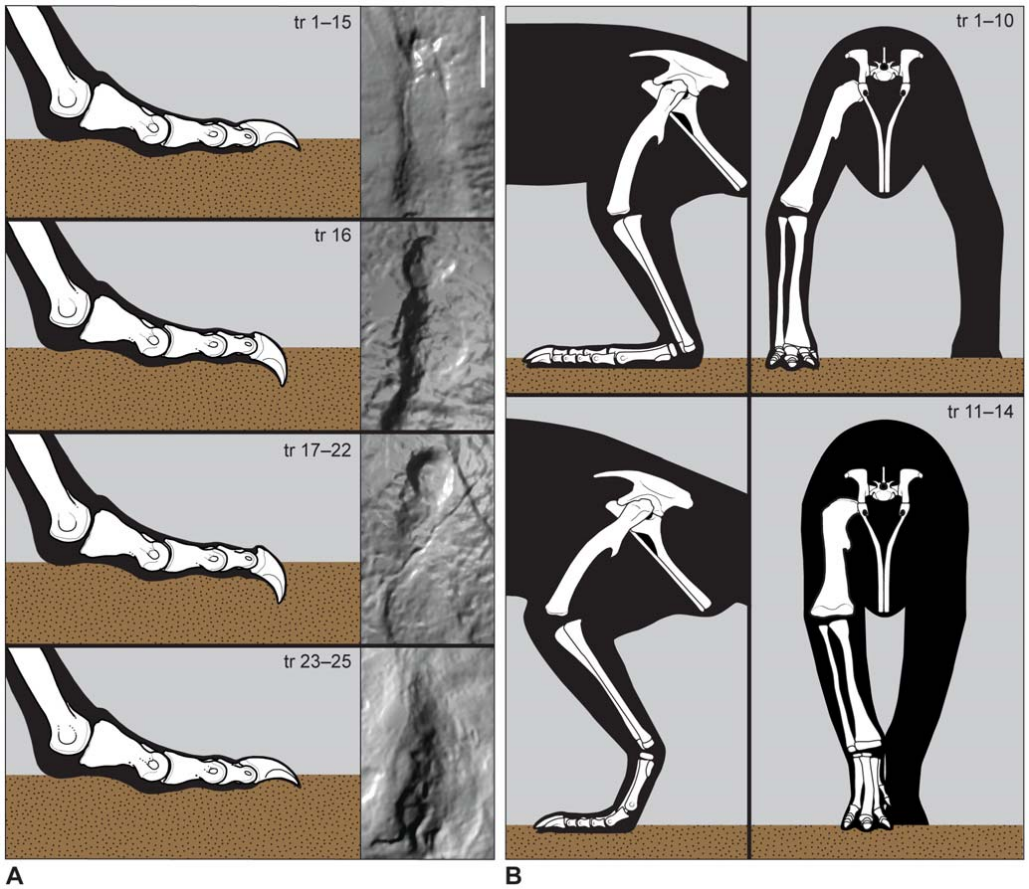
Dynamic locomotor adjustments to paleosurface heterogeneity. A, “flexed-ungual” locomotion in the *Grallator* trackway, which is attributed to a theropod. Images at right show the impression of terminal end of the third pedal digit in tracks (tr) 5, 16, 17, and 24. Differences in the shape of terminal impression reflect deeper penetration of the ungual into the substrate, as shown in corresponding schematic interpretations of digit III at right. In the first and last panel, the tip of the ungual makes a narrow, pointed impression. In the middle two panels, the ungual has penetrated deeper into the substrate so that its base makes a rounded impression at the surface. Silhouette morphology and proportions based on *Allosaurus*. B, postural changes in *Anomoepus*. Panels show our interpretation of limb posture during plantigrade, wide-gauge locomotion (top), and digitigrade, narrow-gauge locomotion (bottom) in lateral (left) and anterior (right) views. Silhouettes are based on limb proportions and skeletal morphology common to basal ornithischians [28]–[34].

Few noticeable changes are apparent along the one definitive theropod trackway preserved at Moyeni, despite substantial differences in substrate. The trackway consists of 25 tridactyl pes prints that traverse the point bar top and curve gently down the slope onto the rippled surface at or very near the water's edge (Fig. 2). Digital pads are well-defined, and a typical 3–4–5 phalangeal formula can be inferred for the trackmaker's three weight-bearing digits (Fig. 3A). There is usually no trace of digit V or the metatarsus on any theropod track, unless the animal is sitting [20]. *Grallator* pes prints at Moyeni average 28 cm in length, which is slightly larger than typical *Grallator* prints but intermediate between the maximal pes lengths recorded in Late Triassic and Early Jurassic theropod trackmakers of the Newark Supergroup [21]. Footprint length can be used to estimate a hip height of 1.4 m and a body length of approximately 6–7 m [19], [22], which is close to the size of the coeval southern African theropod *Dracovenator regenti* [23]. Despite differences in consistency and inclination of the substrate, the *Grallator* trackmaker never deviated from a digitigrade, bipedal posture with a parasagittal gait. Pace angles, measured across three successive footfalls (left–right–left or right–left–right) along the length of the trackway, are consistently high (ca. 170°; Text S2). In contrast, stride length was affected by changing topography, decreasing to 75% maximum stride length over the last 7 steps as the trackmaker left the flat surface of the bar and descended the slope onto the river margin. These shortened strides, which suggest reduced speed, were accompanied by a slight forward shift in body weight, registration of the digit I on the substrate (noted by [3]), and the appearance of subrectangular-to-oval shaped claw impressions on the second and third digits (Fig. 4A). Registration of a digit I trace is usually associated with deep tracks [24], but it occasionally occurs in shallow tracks of the *Grallator*-like ichnotaxon *Gigandipus* from North America, often in association with tail drags [13], [25]. At the Moyeni tracksite, impression of digit I occurs without a tail drag or any appreciable deepening of the track, and it is associated with subrectangular-to-oval shaped claw impressions that we interpret as the result of strong flexion of the second and third pedal claws. In these flexed traces, the ungual phalanx and its keratinous sheath deeply penetrated the substrate and left a trace of its cross-section at the surface. This suggests that the inner three digits flexed together and actively gripped the substrate as a real time behavioral response to a sloping and slippery surface.

**Figure 5:**
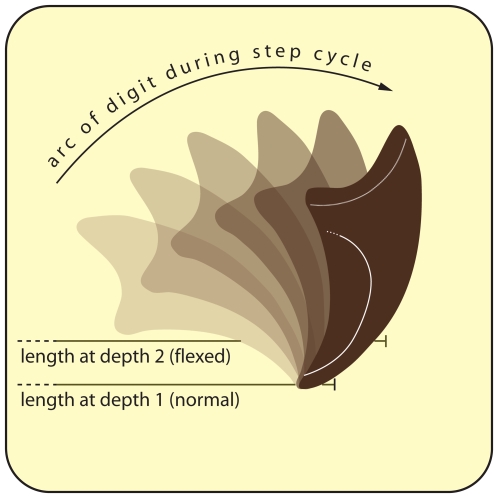
Arc of rotation of ungual during the step cycle. Flexed ungual tracks are longer than typical tracks because the ungual leaves a longer impression when it is submerged within the sediment (compare upper and lower lines).

Two lines of evidence support this interpretation over an alternative explanation for these tracks as a natural consequence of a forward shift in body weight. First, the pre-ungual portion of the pes is shallowly impressed and pre-ungual phalanges are identical in size and shape to previous steps in the trackway. Second, flexion of the second and third ungual phalanges measurably lengthened the second and third digits (absolutely and relative to the fourth digit) compared to those impressed on the bar top (Fig. 4A). Although somewhat counterintuitive, the flexed digits are longer because more of their arc of rotation during the step cycle is recorded in the substrate (Fig. 5).

### Basal Ornithischian Trackmaker (*Anomoepus*)

In contrast to the theropod trackway, the basal ornithischian trackways at Moyeni shift between three distinct locomotor styles, each of which is associated with a different substrate consistency and slope. North American *Anomoepus* has been interpreted as made by a facultative quadruped, based on abundant bipedal and rare quadrupedal tracks assigned to the same ichnotaxon [16], but the Moyeni tracksite offers a unique opportunity to record bipedal and quadrupedal locomotion in a single trackway and to describe the transition between them in detail. This is best demonstrated in the longest *Anomoepus* trackway, which consists of 17 steps that begin on the rippled channel margin and turn sharply up the slope of the point bar and onto the algal-matted upper surface of the point bar (Figs. 2, 3B). *Anomoepus* manus and pes prints along this trackway average 13 cm and 21 cm in length, respectively, indicating a hip height of 1.2 m and body length similar to that of the *Grallator* trackmaker (5–6 m; [19], [22]). Digital pads are not well defined, and manual and pedal phalangeal counts are not known. Although digit I left a trace in some *Anomoepus* tracks, the pes was functionally tridactyl, as indicated by deeper impressions of the internal three digits (II, III, IV), which are large, relatively broad, splayed, and terminate in blunt claws (Fig. 3). When present, manus tracks are more lightly impressed, but they are clearly rotated outwards, pentadactyl, and tipped by rounded unguals.

The first 13 steps in the *Anomoepus* trackway register “wide-gauge” manus and pes impressions that are placed at a distance from the trackway midline (Fig. 2). In these wide-gauge tracks, both digit I and the metatarsus are recorded. The shallow depth of the tracks indicates that the uniform registration of both digit I and the metatarsus is a consequence of a plantigrade postural feature, rather than of deep penetration of the pes into a poorly consolidated substrate [24]. In addition, the shape and size of the metatarsus impressions match those of *Anomoepus* resting traces found elsewhere on the tracksite [11]. Lengthy toe drag marks extending between successive footfalls may have been the result of reduced clearance of the foot during the swing phase of locomotion, indicating that the trackmaker did not “spring up” during the step cycle. Together, the plantigrade hind foot posture and wide-gauge stance suggest that the *Anomoepus* trackmaker adopted a more stable, crouching pose and moved more slowly along the ripple-marked riverbed surface (Fig. 4B). We note here that Ellenberger [3] interpreted these wide-gauge tracks and drag marks as made by a swimming animal, an interpretation we disagree with based on the strong registration of the pes and metatarsus on the substrate (which indicates the animal was supporting its body weight), the regularity of the footfall pattern, and the inferred shallow water in the riverbed [11]. Moreover, the *Anomoepus* trackway lacks features typically preserved in swimming traces, such as shortened prints (which indicates that the animal was buoyed by the water mass) and sigmoid scratch marks of variable length with sediment piled up at their base [26], [27].

The last 5 steps are “narrow gauge” with the limbs positioned along the midline, underneath the body. The transition between the straddling, wide-gauge, plantigrade posture and a parasagittal, narrow-gauge, digitigrade posture was punctuated by a tail impression made during a brief pause (Fig. 2). Pace angulations for the 5 narrow-gauge tracks imprinted on the bank and upper point bar surface are significantly higher than the 13 wide-gauge tracks, all but one of which were made on the rippled surface (186° vs. 107°; Text S2). Additionally, there is no impression of the metatarsus, digit I, or dragged toes on any of the narrow-gauge tracks (steps 14–17). This indicates an elevated, digitigrade pedal posture in which each foot was lifted clear of the substrate during the stride. The final two narrow-gauge steps (steps 16–17) bear no manus prints and demonstrate a third postural change from quadrupedal to bipedal locomotion on the upper algal-matted point bar surface.

The wide-gauge walking recorded in the rippled portion of the trackway indicates a greater range of abduction than implied by *Anomoepus* resting traces preserved elsewhere at the Moyeni tracksite [3]. In resting traces, the implied gauge width is approximately 62% the combined length of the metatarsus and pes; gauge width is twice this in the rippled portion of the trackway (120%). In addition, the differences in gauge width measured along a single trackway allow estimation of abduction angles for the limb. Assuming hindlimb proportions common to basal ornithischians [28]–[34], a hip height of 1.2 m, and taking into account differences in foot posture, we estimate that limb abduction angle ranged between 22° at maximum gauge width (46 cm) and −9° at minimum gauge width (−4 cm), in which the feet stepped across the midline (Fig. 4B). This implies the *Anomoepus* trackmaker was capable of a range of at least 31° of limb abduction. Although it seems likely that most of this mobility was exercised at the proximal, ball-and-socket joint (i.e., hip), some of it may have been taken up at the more distal hinge joints (i.e., knee, ankle).

In summary, in the riverbed, *Anomoepus* maintained a wide-gauge, quadrupedal, gait with a crouching, plantigrade posture. In this crouched position, the animal was apparently not able to fully lift its toes clear of the substrate and left long drag marks that are truncated by the succeeding footprint. On the slope of the bank, the trackmaker transitioned to a more elevated and parasagittal, but still quadrupedal gait. On the stable bar top, the trackmaker adopted a bipedal, parasagittal gait with an upright posture (Figs. 2, 4B). No other *Anomoepus* trackway crossed all three parts of the point bar, but several crossed the inclined slope and algal matted bar top portions. In each case and irrespective of direction of travel, impressions of the manus, metatarsus, and digit I are associated with the inclined slope and absent on the bar top. All *Anomoepus* trackways on the inclined slope and bar top are narrow-gauge.

## Discussion

High-fidelity preservation of long, continuous trackways of early dinosaurs crossing a heterogeneous paleosurface at Moyeni documents real time responses to substrate quality and inclination. This particular set of taphonomic and sedimentological circumstances is rare and allows detailed reconstruction of the locomotor behavior of the trackmakers. Nonetheless, we infer that the locomotor behaviors inferred from the Moyeni tracks are likely to be common to other basal ornithischians and theropods, due to their morphological similarity to contemporaneous North American track-types [4], [14]–[16]. Accordingly, we suggest that the dynamic adjustments left by early dinosaur trackmakers at Moyeni have the following implications for dinosaur locomotor evolution.

Despite the unevenness of the ground, the theropod (*Grallator* trackmaker) did not modify foot posture or adopt a more stable, wide-gauge gait. Although there are examples of theropods adopting a wider stance at low speeds [35] and at rest [20], [36], as well as rare examples of metatarsal traces in deep tracks [24], the vast majority of theropod trackways known to us indicate a narrow-gauge, bipedal gait with digitigrade foot posture [37]. This suggests that theropods were either anatomically incapable or behaviorally reluctant to widen their gait while moving at typical speeds, perhaps because they were able to accommodate surface heterogeneity in other ways, such as the “flexed-ungual” posture observed at the Moyeni tracksite. We suspect that the ability to grip the substrate with the pes was important because it relieved the forelimb from a role in body support. Although seldom recorded in footprints, this feature may have been present in theropods and their immediate ancestors, but absent in ornithischians and sauropodomorphs. This functionality was inherited by descendant theropods close to the bird line, whose ability to climb inclined surfaces using their unguals [38] has recently been implicated in the “wing assisted inclined running” hypothesis for origin of flight [39], [40].

In marked contrast, the *Anomoepus* trackways demonstrate that basal ornithischians possessed a broad range of functional responses to substrate changes. These include changes to trackway gauge (wide vs. narrow) facilitated by limb abduction, foot posture (plantigrade vs. digitigrade) accommodated by ankle flexibility, and the number of supporting limbs (quadrupedal vs. bipedal). The ability of the *Anomoepus* trackmaker to facultatively enlist the front limbs in locomotion in real time foreshadows the three independent evolutionary acquisitions of facultative or fully quadrupedal posture in ornithischian history [41], [42]. This ability has been long inferred for basal ornithischians [43], based on osteological evidence and on their phylogenetic intermediacy between bipedal ancestors and quadrupedal descendants, but it is demonstrated in the Moyeni trackway. Trackway evidence from Moyeni suggests that basal ornithischians were capable of facultative quadrupedalism less than 30 million years after their origin from an obligatorily bipedal ancestor and contemporaneous with the appearance of the first quadrupedal ornithischian body fossils [44].

## Supporting Information

Text S1 Ellenberger translation. This is an English-language translation of two portions of Ellenberger (1974) that name and describe the ichnotaxa discussed the text. The first part is the author's description of *Neotrisauropus*. The second is a lengthier section on *Moyenisauropus*. The original text is in French. Translators: Emile Moacdieh and John Whitlock; editor: Jeff Wilson.
(0.14 MB PDF)

Text S2 Measurements of two dinosaur trackways from Moyeni, Lesotho. Includes measurements of lengthy trackways of the dinosaur ichnotaxa *Anomoepus* and *Grallator*.
(0.07 MB PDF)
