# Supplementary material for: Dynamic Locomotor Capabilities Revealed by Early Dinosaur Trackmakers from Southern Africa
Source: PLoS One. 2009 Oct 6;4(10):e7331. doi: 10.1371/journal.pone.0007331 (PMC2752196; doi:10.1371/journal.pone.0007331)
Supplement: Text S1 — Ellenberger translation. This is an English-language translation of two portions of Ellenberger (1974) that name and describe the ichnotaxa discussed the text. The first part is the author's description of Neotrisauropus. The second is a lengthier section on Moyenisauropus. The original text is in French. Translators: Emile Moacdieh and John Whitlock; editor: Jeff Wilson. (0.14 MB PDF) [file pone.0007331.s001.pdf]

TEXT S1. TRANSLATION OF EXCERPTS FROM ELLENBERGER (1974)

Below is an English-language translation of two portions of Ellenberger (1974) that name and describe the ichnotaxa discussed the text. The description of *Neotrisauropus* (now *Grallator*) appears on pages 18–21 of the original; the description of *Moyenisauropus* (now *Anomoepus*) appears on pages 25–35. The original French text was translated by Emile Moacdieh and John Whitlock and edited by Jeff Wilson. The full reference is:

Ellenberger P (1974) Contribution à la classification des pistes de vertèbres du Trias: les types du Stormberg d’Afrique du Sud (II partie): le Stormberg Superieur-I. Le biome de la zone B/1 ou niveau de Moyeni: ses biocenoses. Paleovertebrata Mémoire Extraordinaire 1: 1–202.

Neotrisauropus deambulator nov. sp. (Type 62; Pl. A-B; Pl. VIII-IX)

Hypodigm (Type): Sandstone slab known as "Moyeni Slab", greenish-white in color, intercalated within the first red sandstones of upper Stormberg facies. The hypodigm includes a complete track (Track Y), well-defined with 26 exposed consecutive steps, representing a life scenario, which will be described below. - A certain number of other tracks are seen elsewhere (Sand bank 250 m to the South-East; deposit of Mokanametsong, etc.)

(a) brief diagnosis:

- type species;
- strict biped, fast, even more "high on legs" than species *N. mokanametsongensis* of the same zone B/1; some future grallatorids will have a similar gait, but without the use of the hallux;
- the hallux touches the ground with its tip on the internal side of the foot a little to the back, more clearly in our species than in the other;
- the median digit III projects farther in front of the lateral digits II and IV than in the related species;
- the external IV digito-metatarsal articular pad is by far the best-printed of the 3; exceptionally, the second digit is weakly supported in this species ;
- the feet, with 3 rectilinear toes, subparallel to the gait and built more gracile, are printed, slender, in an alignment almost strictly rectilinear, as opposed to our other species
- in dimension, exceeding the other species by almost 5/4

(b) Gait and mode of life ("fossilized alive") on the Moyeni Slab

We have the privilege of being able to typologically study our animals within their ethological context. We shall thus follow *N. deambulator* in the finest details of its activity, so as to better understand it. We will forget for one moment that it is an object to take it as a subject.

I. – (It "saunters" initially). The large animal goes up the river's right bank: one sees it following the bank a short distance from the water, that is to say hardly more than 1 meter, on a ground seeming "slightly frozen after a preceding night (?)" (see description of this paleo-phenomenon with *Episcopopus*). It advances along a narrow path in the most precise direction of the term. On this track of practically null width, it goes sauntering with great regular steps of 0,95 m ("valgus steps" of +5° at most), with the three digits close together. - Such is what the first visible steps show us of this long walk.

II. – (It "veers to the right as if to go to the water"). It is now only 0,80 m from the water (extreme limit towards him of the ripple-marks of the small bay or cove "k"). In front of it on its right is the small headland of the bank (headland "j"), route of 3 minor tracks: D coming out of the water, and E and F going down into the water, all three preceding its arrival. Furthermore, the 7th print, abruptly shorter by 0,30 m from the preceding, the has turned resolutely. One would believe that the animal will enter the water; the 3 toes are wide apart perhaps because of the unstable ground.

III. – (It "turns suddenly towards the ground"). - And yet, at this precise point (8th and 9th step), it makes a sudden change of 40° to its left, the toes quite spread (steps shortened by

0,20 then 0,15 m as compared to the normal); then, turning even more to that side to avoid the small bay "i", it resumes its walk with majestic, 0,95 m rectilinear steps,thus abandoning the river (10th to 18th steps). Here it is on the median croup of the island which it goes up lengthily. Near a light dune cord bordering the "lake" side of the island, *N. deambulator* starts a light curve that brings it little by little back to the river below its right (19th to 22nd steps, always 0.95m). The toes are strongly closed .

IV. – (It "goes resolutely to water now"). It left the dorsal of the island and without any hesitation goes this time to water. The 23rd and 24th steps slow down (0,75 m). - With the 25th step (right), it crosses the bank at the end of the very light headland "b", its toes splayed, whereas with the 26th it disappears into the water, toes spread apart to the extreme: the claws of the toes II and IV sink into the ripple marks as if to start a strong push counter-current to the river. One sees a small, muddy splash at the back of the foot. Here the steps of *N. deambulator* join the other tracks of the island, although on top of the majority of them, therefore impressed slightly more recently.

(c) other tracks. It is in a context definitely less varied, and with *Moyenisauropus natator*, that one will find *N. deambulator*, on the South-Eastern sand bank (250 m away). -But the environment of Mokanametsong, where also its steps carry, proves to be of another order. This animal appears driven "to migrate" or better still “to visit" very diverse places, as we will see below.

(d) Detailed diagnosis of the species (description).

To the characters defined above (generic diagnosis and our brief diagnosis), are added many specific precise traits (measurements):

- I. PES - measurable length of the foot 260 mm. Length of the internal lateral support, of the first toe on the ground, 50 mm, sometimes reduced to the proximal articulation or the only ungulo-phalangeal pad; length of the distal support of second toe, 135 mm, or 167 mm with the digito-metatarsal articular pad (slightly printed on 50% of the prints); length of the distal support of the third toe 167 mm, or 195 mm with the digito-metatarsal articular pad (same remark as before); length of fourth toe with the proximal articular pad, 178 mm. Length of fifth toe: ?

- the skeleton of the foot, reconstructed, on the numerous track data, reveals for this impressive animal a phalangeal formula of rather traditional type: 1-2-3-4-(0). The measurement of the toes, made on the reconstitution of the skeleton, can be estimated (with a very small margin of error, - claws included) at: I, 60 mm; II, 155 mm; III, 190 mm; IV, 163 mm; V?. The claws themselves measure, following the analysis of the track: II, 55 mm; III, 47 mm; IV, 43 mm; the strongest being that of the second toe; that of the hallux could measure 25 mm. Of the metatarsals, functionally we perceive only the distal extremity: III is the longest, IV is a little shorter than II. Metatarsal I, much shorter, is practically under II, as in carnosaurs. In contrast, the very tightened and parallel bundle that the metatarsals form brings this shape closer to the foot of coelurosaurs, from which it differs in the exceptional length of the median toe III (?).

- the projection of the third toe, in front of the tips of the side toes II and IV, reached 88 mm, a considerable amount!

- the width of the foot, between the extremities of claws II and IV, reached an average value of 175 mm, but goes down to 160 mm by tightening of digit IV on dry ground, to rise to 220

mm in water, which represents very high possible lateral mobility for digit IV, and to a lesser extent for digit II. The short digit I, perpendicular to the others, is only slightly mobile, its claw capable of moving forwards a few millimeters at most (10 mm maximum).

- total divarication of the toes, II-IV, varies from 33˚ on land, to 63˚ in water (II-III, 19˚–33˚; III-IV, 14˚–30˚), which shows a possible variation which could practically go from simple to double in this spacing, with the foot flat on the ground. Digit I is always directed 83˚ with respect to the digit III (a value that drops only once to 75˚, in the fifth step, track Y, near the water).

- the mobility of the toes, in the vertical direction, is attested by a precise study of the small transverse folds of skin under the toes: the folds are obscured under digit I, but are very strongly pronounced for the toes of II, III, IV, of which all the phalanges are flexible downwards, with 2 small folds associated with the claw, especially that of the third toe. The distalmost inter-phalangeal articulation of each finger is accompanied, strikingly, by 2 to 3 fine folds that are still distinct towards the internal edge of these fingers (and ornamenting a slightly oblique constriction separating, on the ground, the lobes of the digits). The third toe, particularly mobile (with a powerful flexor surface), also displays the same phenomenon in its most proximal articulation : the small folds, normally measuring only 8 to 10 mm in length, here attain 20 mm in visible length. Careful analysis shows that, as in the manner of walking birds, these folds of the skin were done, in *Neotrisauropus*, so much a little in front of the osseous articulation (slightly distally then)? - the foot, raised, would folded up towards the interior.

- the cutaneous covering of the feet bears weak "alignments" a few millimeters long, and reticulated.

II MANUS - the hand is unknown, even when the animal hesitates or when it enters the water (a biped in the strictest sense of the term, *Neotrisauropus deambulator* very badly evokes carnosaurs, coelurosaurs or thecodontosaurids. The autopod radically separates our animal from the group of carnosaurs, as well as the pace of its metatarsus for example). It is regrettable that the hand is hidden from our eyes. Only in a later form of Trisauropodian will we be able to observe the small, special, pentadactyl hand.

III TRACK - the stride measures 1,90 m (step: 0,95 m), but can drop to 1,48 m (step: 0,74 m) in wet areas. The ped. angulatio. is almost null, 3˚–5˚ (hardly valgus steps). The steps are done on a track with null spacing, or little is necessary(spacing oscillates between 0,02 and 0,03 m), and this whatever the length or the brevity of the steps. From where, in animalist vision, a high-legged animal. Even in water (spacing 0.07 m), it still appears to benefit from its high stature (ped. angulatio: 10? . . .). - The tail does not come to be used as support on the ground no more than the hands, even at the most delicate points of the route - turnings, or descent into water, - unlike the 'coelurosaurs', etc.

(e) Plesiotype: On the nearby slab (other islet), there are 4 or 5 trackways which appear identical to that of our type (after a small red loessian intercalary formation). - Within the red loessian pond of Mokanametsong, one finds *N. mokanametsongensis*, whose analysis will be made below, squatter, "larger", more "aggressive" of pace within a slightly different faunal environment.

(f) Derivation of the name and discussion. Named regarding its beautiful and noble step, from the Ciceronian verb *deambulare*: "to go for a walk, and to walk a long time"; such as in Moyeni in the case of our animal with the elongated legs, surveying its sand bank from

beginning to end, and entering its river at a point selected by itself. A discussion on: the general model of *Neotrisauropus deambulator*, the restitution of its osseous frame, its zoological place, its behavior, by applying data of Myologic and Functional Ichnology (see P. Ellenberger "L'Ichnologie, essai méthodologique", op cit. chap. II to IV) will be treated later on (For the systematic position of Trisauropodians, one may refer to our Course on the Systematics of Archosauriens, op. cit.).

N. deambulator must be approximately 3,20 m long. Its high-legged bipedalism would raise its head to over 2,00 m.

<u>Genus M o y e n i s a u r o p u s nov. gen.</u>

1°) Introduction:

This tridactyl genus with a relatively short median finger, a splayed foot, webbed feet, having a tendency to press evenly on the ground, when at rest: its two metatarsal heels lengthened, its hallux towards the interior, its tail in back, its small hands in front, is rather similar to the ichnogenus "*Anomoepus*" Hitchcock 1848, of North America.

Lull (1953 p. 193) said of "*Anomoepus*":

“Bipedal in gait, the manus impressing only when resting. Pes tetradactyl, digitigrade, with elongate metatarsal segment upon which the animal sits. Hallux half rotated, rarely impressing. Moderately long limbs. Tail marks when the animal slows down, but never dragging when the animal walks normally”.[1]

All these features correspond to *Moyenisauropus*. But they are largely insufficient to determine the beast, and correspond to at least 4 kinds of animals of Connecticut, the others doubtlessly being a little more recent (arkoses of Portland or Upper Newark would represent the end of the Triassic, or better, the Rhaetian, even the lower Lias (?) - cf. Lull 1953 p. 16-17, etc).

2°) Generic Diagnosis.

Complementing the generic characters offered by Lull for his "*Anomoepus*" that are valid for many types of animals at rest (see for example Lull, ibid p. 205-208, and his *Sauropus*, which is almost certainly part of an entirely different group), we give the following precise generic diagnosis for the genus *Moyenisauropus* from the Triassic of South Africa (Upper Stormberg, zone B/1):

I Generic features characteristic of *Moyenisauropus* at rest:

- 3 median fingers II-III-IV: spread when walking, close together when at rest (adduction)- (this fact remains undetermined for "*A*", see Lull fig. 60, etc).

- digit 1 (hallux): appears much more frequently in tracks of our type-species than in the American forms; sometimes in its entirety (which is never seen in Connecticut).

- nature of the fingers: the cutaneous folds, the direction of their flexion, their torsion, their descent to the ground when aimed there, give capital indications on the nature of the foot (features often ignored in Connecticut).

---

[1]Ed: Ellenberger translated Lull’s text into French and added additional text of unknown provenance about tail drags. Here we provide Lull’s original text, which forms the majority of what Ellenberger quoted.

- metatarsals: they form an asymmetrical figure at the back of the foot, only metatarsal IV distally touching the ground, unlike metatarsal III and especially metatarsal II, whose supports are only proximal (*Moyenisauropus* differs from *Sauropus* in this regard as well. - See Lull - ibid, fig. 60 and 63; 61).

- hands: small, five-fingered, extremely mobile, sometimes being printed several times when at rest, in a large circle, either in front of the feet inside, or much further outside. (This phenomenon opposes what we have been given for "*A*": see Lull - ibid, p. 192 ss). The hand reveals a metacarpograde tendency in the type-species, an "avian" tendency as we shall see (unknown from Connecticut).

- ischial marks: marks in the shape of 3 small encased crescents, for the type-species (It differs from the circular point of "*A". c.f. intermedius*, Lull ibid, fig. 60), as it does from the little indented circle of *Sauropus* (ibid p. 205-207). - The mark of the sternum (typical at "*A". scambus*, fig. 61) never appears in our species.

- tail: mark almost entire, regular, typical and measurable at each stop (contrary to the "Anomoepodids" whose tail only appears rarely, indeterminately, and only briefly ibid p. 193).

- "muzzle": mark a little lateral to the trackway, of which description and interpretation will be given below, with the diagnoses of the species.

II Typical generic features of *Moyenisauropus* ambulation:

- divarication: when walking, the 3 fingers of the foot, held close together at rest, are spread for stability in a divarication that the nature of the ground will no longer influence, whether "dry" or "wet (it is the inverse of what we saw in *Neotrisauropus* and the Trisauropodians). Only when the animal stops are the toes adducted.

- palmation: the membrane (palmation) which connects the 3 median fingers in all our species, stretches when the animal walks; its traces disappear when the animal decelerates and stops (nothing like it was noted for "*Anomoepus*" in its various forms in North America).

-claws: the presence of claws is noted (and not of "Ornithischian' nails ', as Lull assumed for "*Anomoepus*", 1 S p. 193).

- wobbling: the step shows special wobbling (ped. angle exceeding 20º) with the feet turned inwards, "varus steps" (ped. orientation of -15° - 20º). When at rest the feet are turned outwards (+ 5º).

- the swimming stroke: (type-species): the toes, much more than the fingers, start to enter the ground vertically, the trackway then widens considerably (no swimming animals in Connecticut).

3º) The entity of the new genus *Moyenisauropus*, as opposed to the "Anomoepodids" of Hitchcock and Lull, thus appears very clearly.

The genus *Moyenisauropus*, despite an already advanced speciation, seems striking, considering the polymorphism of the American forms

A typological analysis concerning the genus *Moyenisauropus*, beginning with the type species *M. natator*, will bring us, in conclusion, to classify them within a new family, to be called the "Moyenisauropodids" or, in a paleo-ecological form the "Limnaviens", or “swamp or lake animals with avian adaptations”. For a more thorough systematic discussion, see the end of the typological and bio-ecological study devoted to the type-species.

4°) the name given to the genus.

The name conferred on this small, specific group of Dinosaurs, in our zone B/1, is in homage to the locality of Moyeni, chief town of the District of Quthing in the South of Lesotho, and more still in in recognition of the natives of Moyeni, whose whose amazement and interest were great when the deposit was discovered ("1000 years are... like yesterday when it is over, like yesterday’s night!"). A lucid and constructive interest which has never decreased since...

Moyenisauropus natator nov. sp. (Type 64 ABC; Pl. CD; Pi. XàXVI).

Hypodigm (Syntypes): The greenish-white sandstone slab of Moyeni, intercalated between the first red sandstones of Upper Stormberg in association with *Neotrisauropus, Plastisauropus* and the abundant fauna of the area (see Ellenberger et al. 1964, p. 315-6). The hypodigm includes 15 trackways, of identical appearance (see below), showing all the possibilities of this animal.

(a) Short diagnosis:
- type-species;
- specifically *M. natator* exceeds all the other species of the genus, even *M. natatilis,* by its corpulence, and its more developed adaptation for swimming.

(b) Tracks and fossilized lifestyle restored.

We have the very exceptional advantage to be able to follow this animal, typical of its genus, intimately within its habitat, and witness its diverse actions within their ecological framework. While awaiting the discovery of a skeleton, it rests with to us to draw all possible advantages from a meticulous observation of its frolics and to approach it zoologically at best. The adventures of this animal’s life, which the tracks enable us to witness or take part, may be enumerated as follows: from bipedal swimming, (Track X); a landing upstream (Track Q); clean strokes (Track AW); a "meal" on the ground (Track H); an exit of the river with the hands dangling (Track J); a re-entry into the river for a swim (Tracks L, N, P, M); traveling the river upstream (Track AH), and downstream (Track W). In order to understand the following text, it is important to refer to the large gatefold, as well as the photographs and other documents, which are attached to this work.

I. Track X. "swimming with two feet" Swimming in shallow water, the animal travels the river downstream, coming increasingly into contact with the edges (inlets "e" and "g”):

- ("end of swimming") At the time of the trace (I), the animal, with toes II and III of its right foot, furtively touches the riverbed. Here the hand, dragging, makes an immense rectilinear gash 1,50 m long on the bed with fine ripple-marks, before planting itself there in its turn. (there is a short, curved trail, of 3 fingers each 4 cm apart, right before). The swimming slows down. With the momentum of the body, still half supported by water, the tridactyl foot still hits the ground far in front of the hand, that is to say 0,40 m ahead (not 3) (the manus -- pes pair is strongly reversed!).

-("the animal then takes foot") - What happens next? The strides, becoming increasingly shorter, still reach 1,40 m, but the pes-manus couples cease being reversed: the animal has slowed down. The track, narrow enough when swimming (less than 0,20 m) has become very broad (0,30 to 0,40 m), the feet from this point on making strong trails or parallel gashes sub-rectilinear in shape (steps 4, 5, 6, 7). Where the ripple marks grow weaker nearer to the bank, the animal seems to find its weight, its "gravity". With steps 7, 8, 9, 10, 11, 12, and 13 the rectilinear trails drawn by the feet become increasingly stronger; the animal finally puts its back feet down fully here, with the hallux well extended, sometimes making a strong impression on the ground (10th step). The heel (170 mm to the rear of the digito-metatarsal articulation) makes its appearance from the 10th to the 13th track. The small hands, which one can now distinguish as pentadactyl, follow a long curve (in the form of "S", a reversed "S" for the right hand). The pes-manus step order is restored and the hands gradually plant farther and farther ahead of the feet with each step.

-("the animal starts to raise its hindquarters")-As the posterior strides decrease (1,30; 1,20; 1,10; 1,00 m), one notes that "those" made by the arms remain of the same amplitude (1,30 m; 1,20 m): when the animal slows, its balance shifts and settles on its acropodes, which carries its front end further, ahead of the feet, the chest remaining inevitably still lowered (see the restoration of *Sauropus*, Lull 1953, p. 207). One notes, at the same time, that while the left hand is set on the track, the right hand tends "to cling" to the bank more and more, off the trackway line: the hand pulled on the external fingers (steps ll and 13). To find such a dissymmetry elsewhere, see *Dijaquesopus* and *Episcopopus*, or even *Pseudotetrasauropus augustus* and *jaquesi* (N° 12, 70 and 34-35).

-("the animal raises its front end and walks bipedally").- if there is any astonishing paleo-vision, it increases with the 14th step. On the end of the small headland "h" of the island, the left foot was planted only a few centimeters before the right foot (0,25 cm). The large "swimmer", turned resolutely towards solid ground, is no longer satisfied with the "traction on the fingers of its right hand": it pushes back the ground vigorously, with its left hand with the five short fingers spread out proximally: initially close, then much farther apart, to 1,10 m forwards on its left, which causes it to swivel up to its right, toward the edge of the bank.
By the 15th step, it has thus settled in a strictly bipedal position: one last support of the right hand makes it complete its balance. It advances (steps 16, 17, 18) towards the raised area of the island only on its hind legs, the feet being aligned on the trackway very close together (0,03 m!), like a true biped.

-("Walking on dune sand and probably diving in the lake").-From the 19th to the 21st step, it passes over a large sand dune, where its steps are faded and, as of

probably the 22nd or 23rd step, disappears off to the East, from the very steep bank that dominates the lake. The tail never drags on the ground in Track X, except behind the steps 11-12 (only once)?

II Track Q."a landing upstream". Track Q shows us an end of rather similar "swimming stroke". But here the animal, which "swam" while going up (upstream of the river), lands rather abruptly at the small headland "d' on the bank. Four steps will be enough for him to get up, with strong support from its off-center left hand, and, in the back, of its tail. It then continues its voyage in the direction of the lake, obliquely, its wide track being narrowed to the extreme.

III. Track AW."pure swimming".-Tail marks in deep water can be seen in the lake (Photo Pl V) in "Track AW". Exits of lake, with climbing of the slope, are seen nearby (Track A, blurred a little, going to a 'meal').

IV. Track H."a meal on land". Track H, also subsequent to an exit from the lake and occurring towards the "end of the day" (consecutive spatters, possibly light over-night frost), allows us to discern what was undoubtedly an "evening meal" at this unique place swarming with mud-dwelling worms (tracks of small amphibians can also be found nearby). The 'meal' hypothesis is to be retained).

- "Scene of the meal": the animal, coming out of the lake, stops there a moment (6th and 7th step out of the lake, approximately). Its large feet together (see also the track A) are accompanied to the rear by long marks by the metatarsal heels and the thumbs (hallux) are spread towards the interior. At the back (0,55 m behind the feet), the furtive mark of a ischial callosity in encased crescents that the tail will come to scuff, which also rests temporarily on the ground (length of the support at the back of the feet: up to 1,00 to 1,10 m). The left hand is motionless for a moment - a small pentadactyl hand, circular, clawed; - but the right hand, pressed on the external edge, moves back and forth, while on this side the 'muzzle' (?) extended outside the trackway has plunged into the dry silt as if to snatch (?) something ("a tapering, more or less cylindrical muzzle"?). After this meal-posture, a scenario truly taken from life, the animal straightened up. It pushed off the ground with its hand, and went off from there, the tail obviously no longer touching the ground, towards the river where it embarks for a "crossing" perpendicular to the current, which goes down towards its right (steps 8 to 13). Will it return? The following tracks are much fresher.

V. Track J. "an exit of the river with swinging hands"-Exactly on the same route, but in the opposite direction, we see, on Track J, the return "in the morning" of the animal, or that of a counterpart or friend identical to him. Leaving the deep ripple marks (which washed away its traces?), the animal faces the small headland (j) from which it had descended, previously (the "day before"), on Track H. It makes landfall while drawing itself up only halfway on its feet; during its first steps, its hands with the fingers trailing (especially the internal fingers) draw large arcs in the sand: the arms swing laterally then forwards. These great C-shaped movements (a reversed C for the right hand) contrast with those lengthy "S" shapes which it made when it was half-swimming in Track X. The fine sinuous mark of its tail becomes rectilinear (6th step), then it ceases (7th step); the animal being well on its feet, advances with firm steps on the light

dappling which has slightly obliterated the traces of the "day before" (H), crosses the thin sandy dune to disappear in the lake, opposite of the river (eleventh step). Hallux seen only in steps 1-4.

VI. <u>Tracks L, N, P, M</u>. "a re-entry with the river to swim". - Track 2 shows us a particular manner of springing into the water of the river. The animal, had disembarked from the lake, in the precise place where, previously, the animal of Track H stepped. On the small projecting ledge dominating the precipice of the lake, one sees his trampling (steps 1 and 2); but immediately after, it crossed thin the small dune cord and starts a "descent" in direction of the small cove (i) of the river. By its 8th step, the animal wanted to slow down, bringing its foot (left foot) to the water. But with the ground being undoubtedly inclined and the stop sudden, one sees this foot printed three times in an authentic 16 cm long slide, in the direction of the riverbed. But in what was certainly rather deep water (see ripple-marks), it regained its balance on its right foot, and planted its left foot on the coast (outspread feet, 33 cm apart). Thus with its right foot ahead again and strongly pushed into the water does it spring forward, its body appearing to float above the ripple-marks at the bottom of the Moyeni river, the waves sufficiently high.
Tracks <u>N, P and M</u> start from a place of disembarkment on the slope of the lake, located at 4.50 m farther South that the precedent; star-shaped disembarkment:

Track N shows us, at the river, the same process as Track L, but without the sliding. The animal begins there its floating to the small cove (g); the left and right feet very far from each other, it starts to push off from this position (40 cm = a similar measurement of spacing to that of the end of stroke of Track X).

Track P, quite similar to Track N, diverges from it on its left while approaching the river, which it also reaches the cove (g), but upstream, a little against the current. Both feet start floating at the same time, the left foot pushing more into the mud.

Track M, quite similar to Tracks N and P, diverge from them on their right. The animal starts by turning obliquely for a moment to the North, veering immediately afterwards to its left, at 15°, and accelerating ahead in a straight line towards the small headland (h), downstream from the cove (g). Grazing it from the side, it bends down (9th step), while resting backwards on its fine tail (length: 1 m). It lowers itself some more, scraping the ground furtively, where the ripple marks of the current begin, with its left hand initially (9th step), then its right hand (10th step), and suddenly both at the same time (11th step), the right hand showing that it will turn to its left in this deep water, to ride up the current of the river. This fact is confirmed by the violent kick that it gives one moment after with its right foot, digging in the bed vertically (12th step). After that, one sees nothing any more but virgin ripple marks: it is swimming in the shallow water.

VII. <u>Tracks AH, W and 0</u> (cf. <u>R. and U</u>). "Walk in the river". Another scene of the animal, here: surveying the river by going up the watercourse (<u>AH</u>, with less than 1 m of bank); or going down it(<u>W</u> at 1,50 or 2 m of bank). Track 0 shows penetrating with both feet fully, which is rare: this could be a "second day", a "day later" when the water level had decreased (see below under *M. vermivorus* and remarks of micro-chronology).

VIII. A synthesis view. The Moyeni Slab enables us to discern a great deal of truly exceptional information concerning *M. natator*, its conformation, its adaptations, and even its body measurements.

(c) Synoptic and summary table of the 15 Tracks in the hypodigm of *M. natator*[2], on the island of Moyeni. Tracks A and H go back to just before the "nocturnal frost", the others are from the "following day":

(See the summary table on the following page, page 31).

Track	# steps	½ stride(cm)	Pes L(cm)	Pes l (cm)	Pace ang	Ped ang	Diff. (cm)	Remarks
A	5	from 60 to 30, meal	20	16-17	138	21	40	towards the river, meal before freezing
H	10	43 meal, walk	19(34)	16-20	150	15	30-37	towards the river, complete meal before freezing
J	8	43-44 stop walking, accostage	20	18-17	145	17	30	return to the lake, after freezing
L	11	45 slipping, walking	18	18-20	150	15	32	towards the river, downslope
M	9	55 kick, walk	20+	20	158-150	15(11)	25-33	towards the river, departure swimming
N	10	42 to 54 walk (dry; water)	18-20+	15	135-143	22-18	35	towards the river, hard ground, swimming
O	9	56 to 60 on dry land	20	20-21	160	10	33,34	towards the lake, soft ground,impertur bable walk
P	8	40 to 45 then stop (shore)	18	20	120!	30	42	towards the river, hard ground
Q	8	55 stop swimming bipedally	18+	16-20	121-180	29-2	48	towards the lake, beaching

[2]Ed: Ellenberger wrote "*N. natator*" here but almost certainly is referring to *Moyenisauropus*

Track	# steps	½ stride(cm)	Pes L(cm)	Pes l (cm)	Pace ang	Ped ang	Diff. (cm)	Remarks
R	8	47, 54 in the water	19-17	19	136	22	30	towards the river and swimming
U	3	45 on dry land	18	18	143	18	32	towards the river, hard ground
W	9	57 descending into the river	19-20	17-20	160-135	22(10)	37-50	into the river
X	18	80 (water); 72, 67, 65, 51, 47, 37 (beaching); 55 and 57 (dry land)	20, 17	14-20	75-170	52-5	20 (swim), 35-40 (water) 2 (dry land)	from the river to the lake
AH	9	55 (into the river)	16-20	19-20	-	-	-	into the river
AW	-	tail drag	-	-	-	-	-	deep water of the lake

Total 15 125 (60 meters +)

- Nowhere does *M. natator*[3] keep its feet on dry land for a long time. One constantly see exits or re-entries to the water, with ephemerous transverse passages of the island.
- *M. natator* is found in association on this island, this is capital, with a true biocoenosis of worms, amphibians... (a typical environment).
- Our biped, within the ecological niche that its smaller colleagues contend with it, has, like them, webbed feet, but which seems more rudimentary here (see Tracks 0, X, etc.); it is in addition striking to see our animal, before leaving Track X, following almost rigorously, and for several meters, the route, going back to not long before, of the large crocodile (?), *Episcopopus* (See steps 25 to 40 of this last, pages 53 ss).

(d) Detailed diagnosis. The systematic superposition of each - rather similar - print of the different tracks, allowed:

1. Elimination of anything accidental among the prints;
2. to obtain, by decantation, many essential features of footfalls;
3. to visualize in an increasingly precise way this animal using the appearance of its tracks.

We will complete the diagnosis as follows (total figures obtained):

1.-PES - Length of the foot 205 mm (claws included), with the metatarsal heel included 380 mm. Length of the fingers: of I,.70 mm; of II, average visible distal support 95 mm; III, visible distal support 115 mm, from the metatarsal joint 157 mm; IV, from the metatarsal joint 150 mm; V: ?

[3]Ed: again, Ellenberger here mistakenly wrote "*N. natator*"

- •With the detailed analysis of very many traces of feet made under various conditions by this animal, and with new osseous discoveries made in the levels of this age, we can represent with a rather good degree of accuracy the skeleton of the foot of *M. natator*. Length of the fingers: I, 67 mm; II, 115 mm; III, 150 mm; IV, 122 mm; length of the metatarsals I, approximately 155 mm, II, 195 mm; III, 210 mm; IV, 190 mm; V, 85? mm (according to the print of the heel). The articular folds of the skin under the fingers appear always a little in front of the bony articulations of the phalanges between them; these animals strongly bend their fingers when contacting the ground (see Tracks M, X...).
- •Digit III is the longest digit, but only by 65 mm. Width of the foot between the lateral digits II and IV, 200 mm (170 mm only at the stop for the "meal", feet together; tightening up to 140 mm when swimming, feet pricking the ground). - a palmation, connecting only the proximal part of the fingers, is visible when the foot is spread out (Tracks 0, X). Total divarication of the fingers II-IV, 83° (only 65° when stopped; and much less still when swimming); divarication II-III 40° (30°); III-IV 43° (35°). -The phalangeal pads are marked, cut short, forming rounded lobes; the articular folds are more clearly marked distally, showing a medial finger which is bent towards the internal edge of the foot. The extensors are also powerful, the animal can assume a digitigrade posture (Track S AH, 0, etc). -The pad of the proximal articulation (digito-metatarsal) is quite visible for digit IV, sometimes for digit III, and in digit 1 during resting posture, but never for the digit II, a commonly known difference between Carnosaurians and *Masitisisauropus* (Section II, p.79; p.84 ss).
- •There is a "varus foot". There is simultaneously a "varus step" with-15°, -20° when walking. <u>Ped.orient.</u> increases +5° at rest -In this special case (rest or "meal"), the long heel metatarsal, formed by the very narrow bundle of the metatarsals, is instructive and quite characteristic (170 mm). Metatarsal III appears definitely more sunken, proximally, in the ground than the other metatarsals (which narrowly border it in this special part of the spindle). One sees a palmation much clearer between digits IV and III than between III and II (Track S J, end of X, etc).

II MANUS-Length of the hand, 100 mm (digit I 1.35 mm; II, 40 mm; III, 36 mm; IV, 26 mm; V, 15 mm). Digit III extends 20 mm beyond the other digits. Width of the hand between I and V, 100 mm. Total divarication I-V: 120°. The hand, during ambulation, remain always very open, with fingers widely spaced. At rest it is strongly angled laterally ("valgus steps") (manus orientation: + 30° to 37°), as we have said a relatively avian feature, with a tendency towards a "metacarpograde" posture (X). When half-swimming, the external digits II to V cling to the ground (superficial common flexor?), while the thumb (pollex) is raised (supination). Apparent phalangeal formula: 1 - 2 - 3 - 2 - 1 (?). the fingers of manus are much shorter than those of the Gryponychids (cf. Romer 1956 p. 385), animals that we find on the slab close to the Mokanametsong. It seems that there is a clear tendency, even, for the occasional "carpograde" posture (Track X to compare with Track H).

III TRACKS - Stride, normally 0,90 m (varying from 0,84 to 0,94 m, that is to say 0,42 to 0,47 m); but in water, or moving fast, becomes 1,00 or 1,10 m (1/2 stride = 0,50 to 0,55 m), and exceptionally, while swimming, 1,60 m ("step" = 0,80 m). <u>Ped angulatio</u> from 15° to 18° (in fast movement, this can drop to 10° or 11°; at a slow pace or when climbing the bank, can rise to 22° or even 29° with average 30°).-Spacing, 0,20 to 0,25 m on average (can go down to 0,15 or even 0,02 m when walking fast or special cases (X);

or to increase to 0.37 m when at rest. - 0,35 to 0,40 m when half-swimming, the body apparently being lowered in water and limbs deviating on both sides from the midline (the axis of the track). Manus angulatio: one sees a large possible variety in the positions of the hands; they are posed normally in front of the feet, the arms describing circles, s-curves or straight lines; they can graze the axis of the track (X, 15th step), or imprint far from it (left hand range to 40 cm of the axis, X, ibid). When half-swimming, the anterior spacing reaches 0.55 m.

- One sees marks of scales on many tracks (with the foot, or sometimes with the hand, X, 15th step); the scales are of very little width, millimeter-length, and poorly aligned.

(e) Plesiotype: The Mohaleshoek-Kubake Slab (cf also Moyeni SE, 250 m).

(f) Derivatio nominis and discussion.

I. Name and varieties. *M. natator* thus appears an animal with many aptitudes, flexible and appearing well adapted to its amphibious life. The name comes from the 8 tracks where it appears to be well-suited for swimming. The variety "mosebetsii" (to swim), dedicated to our brother François Ellenberger, known as Mosebetsi or "Active" in the language of Lesotho, appears of great interest (Type 64A). We named v."balnearius" the curved steps on the ground, the swinging arms (Type 64C). Finally we named variety "jejunus" (from fasting, famished) the scenes of squatting, representative of the "meals" made by this animal on firm ground: on the "menu", mud-dwelling worms, possibly amphibians,-the traces of both being found printed instead of the ephemeral "festivity" (?). Could the worms which swarmed there, after the "frost", consequently return later after the passage of our animal (waste? coprolites?) .

II Anatomical vision of *Moyenisauropus natator*. The test of a certain anatomical reconstitution, like that we have modeled for this animal, could be tried using Myological Ichnology on the preserved data (as well as the other approaches that we proposed in our work: "Ichnology, methodological essay" see Chap. III.)

III Systematic placement of *M. natator*. From many points of view, *Moyenisauropus,* in its type-species as in its secondary species, differs from the generic diagnosis of tridactyl biped Anomoepodids from the Triassic. We tackled these systematic problems clearly in our text, already quoted and yet to be published - " Systematics of Archosaurians, in the light of Myological Ichnology, in Gondwana... "

One can say of *Movenisauropus*, and more:

- that it differs from *Neotrisauropus* and *Trisauropodiens* greatly: by often walking low to the ground ("the port"); posture and disposition of the fingers of the feet on the ground and their adduction when in a resting posture (adductions of short duration); their pulp rather thick and fat, a relative reduction and incurvation of the median finger towards the interior; the nature of the not particularly sharp-edged and especially "nonhooked" claws; the amplitude of the hallux directed towards the interior; the distal and especially the proximal support of the long metatarsals; the frequent use of the hands and their rotation towards the outside; the ischial mark; the mark of the tail; the mark of the "snout"; palmation; wobbling on slightly wider trackways, etc. - in all these ways, our Limnavians differ from the Trisauropodians.
- it differs from the "Paleosaurians"; like all animals easily assimilated into primitive carnosaurians, including the Gryponychids (cf also *Aetonychopus*), and the primitive forms

Plastisauropus, *Megatrisauropus*, etc. - *Moyenisauropus* differs from them in many features: by the gait; the shape of the foot, asymmetrical posteriorly; etc.-As for the evolved carnosaurians, with even more powerful claws, one could say at least as much of it, regarding these claws and the symmetry of the foot.

- it differs from Thecodontosauridae. so numerous in the Upper Stormberg, with the subparallel fingers II-III-IV with extremely short median III. - *Moyenisauropus* separates spectacularly from this group in the makeup of the foot (the acropode and the metapodials are shortened there, compact; much longer here). It would be problematic to rejoin our animal, then, with the recent classification of Haubold (1971 p. 83 ss) which joined "Anomoepodids" with slender and slim feet and the "Prosauropods", animals with heavy feet; even if one retained of this unrealistic group only the tridactyl animals and such bipeds as *Thecodontosaurus* and its allies *Massospondylus*, *Gyposaurus*, etc., of Stormberg.

-it differs from the other groups such as ornithopods. - Even if these animals existed in the Stormberg (see *Paratrisauropus*, etc), their characteristics seem to exclude any relationship between our animals and this group to which Lull had, for a time, been tempted to join (Lull 1953 p.192 ss; nature of the claws, etc).

-Finally as for the "Carnavians" that one will see a little lower, like the "Lacunavians" (both from the "pond-waterhole" of Mokanametsong), one can realize, in spite of an avian adaptation that is rather parallel from the ethological point of view, how much and to what extent they are strangers. The first, with *Masitisisauropus*, are small, light, sharp, even aggressive; characterized by their claws, etc, which make them close to carnosaurs, with "feathers" (?) and their strange carpograde or metacarpograde hands with thin, thread-like fingers. The second, including *Trisauropodiscus*, and in our zone B/1 *Masitisisauropodiscus*, are light animals, with long tails and the thin metatarsals of coelurosaurs, with the back legs (and not the front legs) carrying fine filiform digits... bipeds with the hopping appearance of passerine birds.

Ultimately *Moyenisauropus natator*, not hopping, but swimming instead, represents one of the largest forms among the aquatic coelurosaurs engaged in a polyphyletic race towards the "avian rank". If it measures 2,50 m long and if its head rises over 1,50 m high - its spine dropping to a height of 0,80m when squatting-, the fingers are relatively delicate, are carried by particularly lengthened metatarsals, tapering, slender in appearance. With Podokesaurids on one side and Segisaurids on the other, we would readily place them in an important new family, virtually unknown osteologically, named the “Moyenisauropodids”. We believe it might be necessary to put this family in a group that would would be officially christened Limnavians, citing their adaptation to a life like that of the birds of the marshes or the lakes (from the Greek word "limnê" = "marsh", "lake"). - The other species of the genus will still confirm this vision. These animals could possibly have developed within Coelurosauria sensu stricto, perhaps a convergent foray towards this possible "rank".
