# Supplementary material for: Dynamic Locomotor Capabilities Revealed by Early Dinosaur Trackmakers from Southern Africa
Source: PLoS One. 2009 Oct 6;4(10):e7331. doi: 10.1371/journal.pone.0007331 (PMC2752196; doi:10.1371/journal.pone.0007331)
Supplement: Text S2 — Measurements of two dinosaur trackways from Moyeni, Lesotho. Includes measurements of lengthy trackways of the dinosaur ichnotaxa Anomoepus and Grallator. (0.07 MB PDF) [file pone.0007331.s002.pdf]

TEXT S2. MEASUREMENTS OF TWO DINOSAUR TRACKWAYS AT MOYENI, LESOTHO.

Measurements of lengthy trackways of the dinosaur ichnotaxa *Anomoepus* and *Grallator* (see Figure 2) are reported in the table below. Trackway measurements follow standard protocol, summarized in [1]. Stride length is measured as the straight-line distance between homologous points on successive footfalls of the same foot. Pace length is measured as the straight line distance between homologous points on left and right manus or pes prints; successive pace lengths (i.e., R–L–R or L–R–L) form an angle that is measured as pace angulation. Linear measurements were made to the nearest 0.5 centimeter and were measured in situ unless noted; angular measurements were made to the nearest 0.5 degree using a high-resolution map of the trackway surface. Some tracks are no longer accessible because they are no longer visible (e.g., beneath retaining wall) or no longer preserved. These track measurements were estimated to the nearest 1.0 cm or degree based on maps in [2] and indicated by the dagger symbol (†). Asterisks (*) indicate estimates from the map in Figure 2.

In both trackways, the first measured footfall was a left (central columns). The measurement for pace length between tracks 1 and 2 is listed in the same row as "1" and is left-justified within the column to indicate the corresponding pace length was measured from a left to a right foot. The next pace length (i.e., right 2 to left 3), is listed in the same row as "2" and right-justified. Stride lengths are listed in the row between corresponding footfalls, so the stride length between right footfalls 2 and 4 is listed in the same row as "3" and right-justified. Pace angulations involve measurement between

three successive footfalls and are listed next to the middle footfall and justified according to its side (i.e., LRL is right-justified, RLR is left-justified).

***Anomoepus* (basal ornithischian)**								**_Grallator_ (theropod)**					
pace angle (degree)		pace length (cm)		stride length (cm)		track		stride length (cm)		pace length (cm)		pace angle (degree)	
RLR	LRL	L→R	R→L	L→L	R→R	L	R	L→L	R→R	L→R	R→L	RLR	LRL
—		80.0			—	1			—	95†		—	
	118.5°		84.0	155.0			2	198†			103†		175°†
118.0°		77.0			148.0	3			193†	89†		174°†	
	127.0°		83.0	151.0			4	—			95†		164†
121.5°		78.0			149.0	5			159.5	72.0		141.0°	
	137.0°		70.0	154.0			6	135.5			68.0		133.5°
128.0°		83.0			138.0	7			153.0	85.0		173.5°	
	117.0°		87.0	146.5			8	178.0			95.5		167.0°
109.0°		71.0			116.0	9			184.5	90.5		168.5°	
	110.0°		58.5	117.0			10	180*			92*		176°†
104.0°		75.0*			101.5*	11			188*	98.0		177°†	
	96.0°		42.0*	132.0			12	190.5			95.5		170°†
105.0°		50.0*			78.0*	13			194.5	95.0		162.0°	
	186.0°		60.0*	118.5			14	188.5			94.5		172.5°
189.0°		55.0*			119.0	15			190.5	98.0		170.5°	
	184.0°		60.0*	117.0*			16	205.0			109.0		173.0°
—		—			—	17			199.0	93.0		166.0°	
							18	—			—		172°†
						19			189.0	—		157°†	
							20	—			100.0		169°†
						21			167.5	75.5		141.5°	
							22	158.0			86.0		164.0°
						23			158.0	77.5		154.0°	
							24	149.0			73.0		168.0°
						25			—	—		—	

LITERATURE CITED IN TEXT S2

1. Thulborn RA (1990) Dinosaur Tracks. London: Chapman and Hall.
2. Ellenberger P (1974) Contribution à la classification des pistes de vertèbres du Trias: les types du Stormberg d'Afrique du Sud (II partie): le Stormberg Superieur-I. Le biome de la zone B/1 ou niveau de Moyeni: ses biocenoses. Paleovertebrata Mémoire Extraordinaire 1: 1–202.
